# Analysing how AI-powered chatbots influence destination decisions

**DOI:** 10.1371/journal.pone.0319463

**Published:** 2025-03-10

**Authors:** Miguel Orden-Mejía, Mauricio Carvache-Franco, Assumpció Huertas, Orly Carvache-Franco, Wilmer Carvache-Franco

**Affiliations:** 1 Facultat de Turisme i Geografia, Universitat Rovira I Virgili, Carrer Joanot Martorell, Vila-seca, Spain; 2 Universidad Bolivariana del Ecuador, Durán, Ecuador; 3 Department of Communication, Universitat Rovira i Virgili, Tarragona, Spain; 4 Universidad Espíritu Santo, Samborondón, Ecuador; 5 Facultad de Ciencias Sociales y Humanísticas, Escuela Superior Politécnica del Litoral, ESPOL, Guayaquil, Ecuador; Shandong University, CHINA

## Abstract

This study aims to explore the role of destination chatbots as innovative tools in travel planning, focusing on their ability to enhance user experiences and influence decision-making processes. Based on the Technology Acceptance Model, Enterprise Content Management, and Information Systems Security models, the study examines the psychological, emotional, and technological factors that drive user satisfaction, continued use, and intention to visit a destination. Understanding these factors is crucial for improving chatbot design and optimizing their implementation in the tourism industry. A total of 312 responses were collected from university students who regularly engage in tourism-related activities. The survey employed a structured questionnaire with items measuring information quality, user satisfaction, perceived enjoyment, usefulness, and behavioral intentions using a 7-point Likert scale. Structural equation modelling [SEM] was used to analyze the relationships between constructs, allowing us to evaluate the validity and reliability of the model. The results reveal that information quality positively enhances user satisfaction, perceived enjoyment, and perceived usefulness. Moreover, perceived enjoyment and usefulness are critical psychological and emotional drivers influencing users’ decision to continue utilizing chatbots. Additionally, the analysis highlights the intention to continue using destination chatbots as a strong predictor of tourists’ intention to visit the destination. The findings contribute to the theoretical understanding of technology acceptance and user behavior in tourism, while providing practical insights for destination managers and developers to enhance chatbot features and improve traveler engagement.

## 1. Introduction

Chatbots are software programs that use natural language processing [NLP] and machine learning [ML] technologies [1] to understand human language and provide valuable answers to user questions and requests. These agents can reduce response time to user requests [2] and provide user information 24 hours a day with uninterrupted customer service, essential factors to improve user satisfaction [3]. For this reason, they are increasingly used in different industries and sectors [4].

In the tourism sector, companies and destinations use chatbot dialogue systems to communicate and improve efficiency in the management of tourism services [5]. This technology generally provides users with relevant information, advice, and critical support in the tourism industry [6]. The implementation of chatbots in the tourism field has been steadily increasing [7]. This growth is driven by the sector’s digitalization following the pandemic, its interconnected nature, and the need to effectively manage user queries and requests [8].

In hospitality, chatbots help guests book rooms, do the check-in and check-out, request services, or provide all kinds of information [9]. In restaurants they are used to make reservations, order the menu, or resolve questions [10–13]. In the field of transportation, airline companies use chatbots to meet flight reservations [14] tourist destinations use them to provide information and transactions to tourists that generate satisfactory tourist experiences and a good image for the destinations [15,16].

But despite the rapid and growing implementation of chatbots in the tourism sector, academic studies on them are still scarce [17]. Some have demonstrated the benefits they provide in productivity, profitability, and customer satisfaction [18] and have shown that they generate satisfaction among tourists [19], while others are beginning to demonstrate that they also generate negative impacts on users regarding self-efficacy, accountability, and self-identity [20].

While chatbots have demonstrated productivity and customer satisfaction benefits, studies focusing specifically on their use in travel planning are scarce. Existing research primarily addresses chatbots’ impact on hospitality and destination management. Still, there is a notable absence of studies examining how these tools influence travel planning and destination selection. Furthermore, concerns remain regarding chatbot interactions’ accuracy, reliability, and ethical implications, such as the personalization of services and data privacy.

Currently, tourist destinations are implementing technological agents to be able to offer increasingly personalized communication and services to tourists [21,22] that generate more satisfactory tourist experiences and a better destination image [23]. For this reason, studies have begun to appear that have demonstrated the impact of chatbots on tourist satisfaction, the creation of destination image, and on future visit intention [15,16].

Chatbots can help tourists plan their trips by providing information about services and tourist attractions. Technological tools that increasingly influence travel planning and decisions about the destination to visit, and there are studies on them. There are travel recommenders who are beginning to use AI in travel planning [24,25], technologies such as Skyscanner that generate a tourist experience from the moment of selecting the destination [26], virtual technologies such as AR or VR, which show virtual content, or GPT that offers tourist information about destinations, and influence your decision to visit [27,28]. However, there are no studies on the usefulness of chatbots in travel planning and the decision to visit destinations.

Thus, the research gap in this study lies in exploring chatbots as a tool for travel planning and their impact on tourists’ decisions to visit destinations. Previous applications of TAM, ECM, and ISSM models in tourism technology have not been sufficiently extended to chatbot interactions within the context of travel preparation. There is a need to assess how these theoretical frameworks can effectively evaluate user acceptance, satisfaction, and the intention to visit destinations based on chatbot interactions.

This study will improve knowledge about the role of chatbots in travel planning, the antecedents of user satisfaction, and their effect on visit decisions. Additionally, it will guide DMOs, marketers, and chatbot designers in the development and adoption stages. This study aims to assess the effectiveness of TAM, ECM, and ISSM in understanding user acceptance and utilization of chatbots for planning travel itineraries. Therefore, we consider:

RQ: To what extend do TAM, ECM and ISSM provide a reliable framework for analyzing the acceptance of chatbots for travel planning?

## 2. Theoretical foundations

### 2.1. Technologies and travel planning

While previous similar studies have analyzed the influence of chatbots in various fields and sectors such as commerce [29,30], medicine [31,32] or education [33], among others, studies in tourism are still incipient and no one the influence of this technology in travel planning.

Technological evolution has facilitated travel planning in recent decades, and academic studies have focused on analysing how diverse technologies are used and what functionalities they have in this planning. Initially, studies were carried out on information technologies and social media [34,35]. Fotis et al. [34] demonstrated that social media are essential tools influencing tourists’ travel decisions and that user-generated content is perceived as more trustworthy than official tourism websites. Xiang et al. [36] also demonstrated the growing use of social media in travel planning. Similarly, Huang et al. [37] highlighted the importance of travel websites, social media, and smartphones in travel planning and tourist satisfaction. Subsequently, other types of smart technologies, such as VR and AR or travel recommenders, were studied [38]. Disztinger et al. [39] and Gibson and O’Rawe [40] demonstrated the importance of VR in promoting destinations and generating satisfying pre-experiences that influence travel planning and decisions. Similarly, Ahmad et al., [41] also analyzed AR during Covid and demonstrated its influence on intentions to travel to destinations in the future. More recently, AI in its various forms [chatbots, ChatGPT] has also been studied in travel planning [42]. Shi et al. [42] investigated the impacts of systematic and heuristic cues on travellers’ cognitive and emotional trust and its influence on travel adoption intention toward artificial intelligence AI-based recommendation systems in travel planning. Elizalde-Ramírez et al. [43] demonstrated the importance of AI planning models to offer quality recommendations fully adapted to the needs of tourists when planning trips. Recently, various studies are also emerging that demonstrate the influence of GPT Chat on travel planning [28,44,45]. However, studies on chatbots in travel planning are lacking.

Various studies that have analyzed the use of technologies in travel planning have focused on technological acceptance, applying the Technology Acceptance Model [TAM]. However, the TAM is more common in studies during the trip than exclusively in planning. Some studies analyze the use of user-generated content [46,47] in social media [35,48] in travel planning. Others focus on different technologies such as VR [40] or AR [41] or the use of personalized location-based mobile tourism applications [49], but studies on chatbots that apply TAM are also scarce [50] and not focused on travel planning.

### 2.2. Hypothesis development

#### 2.2.1. Information quality.

Information Quality [IQ] is one of the essential quality factors to measure the success of an information system [51] and generates a positive impact on user satisfaction [52]. In this study, informativeness (akin to information quality) is described as “the tourist’s perception of obtaining relevant, reliable, and high-quality information from the chatbot during a conversational session.” This is identified as the primary factor influencing user satisfaction with chatbots p. 2856 [16] IQ can be defined as the accuracy, format, completeness, and currency of information produced by digital technologies [53]. The DeLone and McLean model [54] postulates that the quality of information stimulates user satisfaction. User satisfaction refers to the subjective evaluation of the multiple experiences generated by the user through an information system [55]. Access to sufficient, accurate, truthful, updated, and reliable information determines customer satisfaction [56]. In the case of chatbots, information quality refers to the accuracy and relevance of the answers provided by the chatbot based on the user’s questions and needs. For this reason, we consider:

H1: Information quality of chatbots positively influences perceived usefulness.

H2: Information quality of chatbots positively influences user satisfaction.

H3: Information quality of chatbots positively influences perceived enjoyment.

#### 2.2.2. Perceived usefulness.

Perceived Usefulness [PU] represents users’ perceptions of the expected benefits of using an information system [57]. PU is a key construct in technology acceptance theory [TAM], which is used to understand how users perceive the usefulness of a technology and how this influences their usage behavior. In chatbot contexts, PU refers to the belief that using a chatbot can improve the efficiency, effectiveness, or user satisfaction and experience in performing a specific task.

In the case of chatbots, the perception of usefulness can influence the probability that a user will use and value them positively. It was shown that PU establishes a positive relationship between satisfaction and intention to remain in a technological information model [58], and subsequent studies reinforced this relationship [59–61]. For example, if a chatbot is perceived as being able to provide quick and accurate responses to users’ questions or requests, this may increase willingness to use it. On the contrary, if a chatbot is not perceived as capable of providing valuable answers or is not easy to use, the perception of usefulness is low, which could lead users not to use this tool. Therefore, users who perceive a high level of usefulness in a chatbot are more likely to use it and have a satisfactory experience than those who perceive low usefulness. With this background, we believe that:

H4: The perception of the usefulness of chatbots positively influences user satisfaction.

H5: The perception of the usefulness of chatbots positively influences usage continuance intention.

#### 2.2.3. Perceived enjoyment.

Perceived Enjoyment [PE] is the enjoyment that users perceive of a technological tool, and Melián-González et al. [62] found that enjoyment has a significant impact on chatbot usage intention in travel contexts. De Cicco et al. [63] found that Millennials’ attitudes toward chatbots are positively predicted by Perceived Enjoyment with the chatbot.

Perceived enjoyment reflects intrinsic motivation because it is associated with the pleasure derived from the activity itself [16]. Consequently, it plays a crucial role in determining user satisfaction and technology acceptance [64]. Technology is sometimes used for its entertainment and enjoyment value rather than for performance enhancement [65]. Some examples in the context of technology promote prolonged use for leisure purposes rather than productivity [66]. Thus, there is evidence that perceived enjoyment predicts satisfaction and continuance intention in technological environments, particularly in chatbot applications [67,68]. Therefore, we propose that:

H6: Perceived enjoyment of chatbots positively influences user satisfaction.

H7: Perceived enjoyment of chatbots positively influences usage continuance intention.

### 2.3. User satisfaction, usage continuance and destination visit intention

The relationship between information quality and user satisfaction with chatbots has been demonstrated in several studies. For example, in mobile banking, satisfaction with the tool also positively and significantly affects user continuance intention [69].

Ashfaq et al. [70] argue that IQ is essential in developing chatbots for customer service and positively affects user satisfaction and continuity intention. In the same way, Hsiao and Chen [70] analyzed service quality, trust, and satisfaction to predict users’ intention to continue using chatbots to order food in restaurants, and the results showed that satisfaction indeed had significant direct effects on the intention to continue using chatbots. Therefore:

H8: User satisfaction positively influences the intention to continue using.

Previous studies have shown the importance of DMOs’ online platforms [websites and social media] in creating the destination image and destination visit intention [71]. Likewise, studies on smart technologies [72,73] and tourism applications like AR [74] or VR experiences [75] or Live streaming [76] have also shown that certain technology factors increase usage intention and destination visit intention. It has been demonstrated that using smart tourism technologies increases destination visit intention [77].

In the field of chatbots, social presence and imagery processing have been shown to influence chatbot continuance intention [78]; and in tourism chatbots, a study on online travel agencies chatbots showed that understandability, reliability, assurance, and interactivity positively influence chatbot continuance usage intention, although technology acceptance moderates this influence [79]. Similarly, Pillai and Sivathanu [50] showed that ease of use, perceived usefulness, perceived trust, perceived intelligence, and anthropomorphism influence usage intention in tourist chatbots, while technological anxiety moderates this effect. A study of food-ordering chatbots showed that anthropomorphism, service quality, and problem-solving are the aspects that influence user satisfaction and usage intention [70].

In destination chatbots, Orden-Mejía and Huertas [80] analyzed the effect of chatbot usage intention on the formation of the destination image, demonstrating that informativeness and empathy are the main attributes that influence user satisfaction and the formation of the destination image and also influence the visit intention. Similarly, Tosyali et al. [15] showed that the informativeness of the chat affects the destination image and the visit intention. However, it is necessary to continue analyzing the factors that motivate the continuance of chatbot usage intention and its relationship with destination visit intention. Therefore:

H9: The destination chatbot’s continued usage intention positively impacts tourists’ visit intention.

## 3. Methodology

The present study was ethically approved by the Research Dean of the Polytechnic University ESPOL with code FCSH-14.2021. Informed consent was requested in writing from the interviewers at the beginning of the questionnaire. The data for this research was collected from responses from students from the Polytechnic University ESPOL who experimented with the QuitoGuide chatbot. The selection criterion for the students was based on their attendance in classes during the semester in which the study was conducted and their enrollment in the Faculty of Social and Human Sciences, specifically in the Bachelor’s degree program in Tourism. These students were chosen for their academic background and experience in tourism-related topics, factors that were essential for evaluating the chatbot ‘QuitoGuide’ in a relevant tourism context.

This conversational AI provides information about tourist attractions, hotels, restaurants, the weather, statistical data, or the history of Quito, Ecuador, among other functions. Quito, also known as the capital of the center of the world due to its privileged geographical location, has played an essential role in promoting and expanding tourism in Ecuador, attracting numerous domestic and international tourists. The city was awarded at the World Travel Awards as South America’s Leading City Destination 2022 in recognition of its excellence in the tourism sector [81]. In addition, the architectural and artistic treasures made Quito Cultural Heritage of Ecuador and Cultural Heritage of Humanity by UNESCO for having the most extensive, best preserved, and least altered Historical Center in Latin America [82].

The chatbot analyzed, QuitoGuide, was a finalist in the Chatbots Tourism Awards 2020, awarded in Madrid, Spain. Access to the chatbot can be done through the Facebook messaging interface. The sample was collected between January and February 2022 through convenience sampling, a technique used in tourism and chatbots, e.g., Sitthipon [83] and Yoon and Yu [84].

### 3.1. Data collection

The explanatory study relied on a quantitative method because it focuses on objectives and measures them through actions and opinions. A survey strategy was used for data collection via pre-designed questionnaires. Regarding the sample size, there is evidence suggesting that 100 cases are sufficient when there are three factors with three or four indicators each. However, it is also recommended that the sample size should be at least around 200 cases, even with high communalities or uniqueness, or well-determined factors. In our study, we analyzed 284 cases.

In this study, we employed a convenience sampling method for data collection, which involves selecting participants based on their accessibility and proximity to the researchers. In our case, university students attending virtual classes were readily available and easily accessible through the Microsoft Teams platform. Given the hybrid academic environment during the COVID-19 pandemic, this method allowed us to efficiently engage participants without disrupting their regular class schedules. Although the sample was not randomly selected, the students provided valuable information as they could represent potential tourists interacting with the chatbot in a realistic scenario.

The data collection method was conducted in three phases. First, prior to requesting permission from several instructors to take a few minutes of their class to conduct the experiment, we entered the Microsoft Teams platform to contact students remotely because the university at the time of the experiment maintained a hybrid modality [virtual and in-person classes] because of COVID-19. Once in the digital classroom, we present the study without revealing its objectives to avoid bias and explain the characteristics of tourism technologies, including chatbots. Second, we asked the students to enter the link that directed them to the “QuitoGuide” chatbot. Once in the application, they had to exchange messages for 10 minutes, enough time for this type of experiment [66]. Participants were free to interact with the application, imagining their intention to visit the city of Quito. We emphasize that the university is located on the coast of Ecuador, 420 km away from Quito. With this, we consider having obtained a representative sample of the tourist population. Third, after the human chatbot conversational session, students had to access a link to complete an online questionnaire about their experience with the chatbot. The questionnaire was developed using Google Forms, and quality mechanisms were considered to ensure that each user only submitted the form once. Finally, the participants were thanked for their collaboration, and we said goodbye. The entire procedure lasted approximately 30 minutes on average. In the end, we obtained 312 responses between men and women. However, 28 were discarded for having missing data, outliers or being an outliner.

### 3.2. Measurement

The questionnaire design comprised multiple items and constructs adapted from previous studies. To measure the indicators, we used seven-point Likert-type scales. We measured information quality using seven items adapted from [85]. The scale developed by [69] was used to measure perceived usefulness. Perceived enjoyment was examined with four items used in the instrument developed by [86]. User satisfaction was measured using a five-item scale from [63,86]. The continuance intention dimension was examined with five items from [67]. Finally, the intention to visit destination [IVD] factor was evaluated based on five items adapted from [87] (see Appendix 1).

### 3.3. Data analysis

A partial least squares structural equation modeling PLS-SEM model was used. Variance-based partial least squares structural equation modeling [PLS-SEM] is considered the ideal data analysis method to simultaneously evaluate all structural paths between variables in a conceptual model. In addition, it can achieve a higher statistical analysis level than others based on covariance [88]. For example, it maximizes the variance of endogenous constructs [89] and can handle non-normally distributed data [90]. Additionally, it is effective in estimating complex models with a small sample size while ensuring adequate statistical power.

The study strictly followed the two-step approach of PLS-SEM, inspecting the measurement model before examining the structural model [88]. Additionally, common method bias, mediation, and the predictive power of the model are reported [89]. Finally, model fit indicators and the coefficient of determination R² are presented.

In the first step, the measurement model is created, where the scores of latent variables and the loadings of indicators are estimated through iterative steps [63]. Furthermore, reliability and internal consistency indices Cronbach’s Alpha and Composite Reliability, as well as convergent validity AVE: Average Variance Extracted] and discriminant validity [Fornell-Larcker and HTMT: Heterotrait-Monotrait Ratio, are reviewed. In the second step, the bootstrapping technique is applied to evaluate the significance of the structural path coefficients of the model. Additionally, to determine the effect size or the magnitude of the impact of a predictor on a dependent variable within a structural model, the f² is reported.

Common method bias is a drawback to the extent that exogenous and endogenous variables are collected from the same respondent [91]. To ensure that CMB is not a severe concern in this study, we ensured that the questionnaire items had simple and concise language. In addition, we informed the participants that their anonymity was guaranteed and that there was no right or wrong answer to each statement they had to answer. Finally, we used the maximum likelihood method in Harman’s single factor test [92] using SPSS. The results reported that the common variance explained by the single factor is 48.58% below the threshold of 50% of the variance [93]. Hence, this type of bias is not a serious concern.

WarpPLS 8.0 was used for structural model analysis, which allowed us to evaluate the relationships between constructs and determine the validity and reliability of our model. WarpPLS 8.0 is a robust tool for structural equation modelling SEM, particularly useful for handling small samples and non-normal data, ensuring precise estimation of model coefficients.

## 4. Results

### 4.1. Sample description

The general sample [N =  284] included mainly women [n =  204; 71.8%] and a smaller number of men [n =  80; 28.2%]. Most participants [n =  259; 91.2%] were between 18 and 25 years old. In the importance ranking, students exchanged messages with the chatbot on restaurants, tourist attractions, hospitality, and activities to do and visit.

### 4.2. Inspecting the measurement model

Six constructs were involved in the model that used partial least squares structural equations. The evaluation of the measurement model must meet both reliability and convergent and discriminant validity [94]. The indicator loading must be greater than 0.7 for the reliability to be considered acceptable [91]. The results in Table 1 show that all items are above the suggested threshold; Hence, the criterion is met. Cronbach’s alpha was used to measure the reliability of the constructs with a cut-off point of 0.7 [92] The values in this research range between 0.916 and 0.973, higher than what was proposed by Hair (see Table 1).

**Table 1:** Indicator loadings, reliability and convergent validity.

	Loading	α	CR	AVE	VIF	Skewness	Kurtosis
Information Quality		0.952	0.960	.775	3.03	-1.31	1.38
IQ1	0.859						
IQ2	0.896						
IQ3	0.900						
IQ4	0.901						
IQ5	0.882						
IQ6	0.850						
IQ7	0.873						
Perceived Usefulness		0.957	0.966	0.852	3.25	-0.98	0.71
PU1	0.929						
PU2	0.947						
PU3	0.922						
PU4	0.925						
PU5	0.893						
Perceived Enjoyment		0.951	0.965	0.873	2.90	-1.36	1.96
PE1	0.944						
PE2	0.922						
PE3	0.944						
PE4	0.925						
Satisfaction		0.916	0.938	0.751		-1.21	1.13
SAT1	0.849						
SAT2	0.909						
SAT3	0.922						
SAT4	0.874						
SAT5	0.771						
Continuance Intention		0.973	0.979	0.904	2.72	-0.87	-0.00
CI1	0.957						
CI2	0.954						
CI3	0.947						
CI4	0.945						
CI5	0.949						
Intention to Visit Destination		0.930	0.947	0.782	1.61	-0.93	0.02
IVD1	0.888						
IVD2	0.909						
IVD3	0.882						
IVD4	0.887						
IVD5	0.854						

α =  Cronbach´s alfa; CR =  composite reliability; AVE =  average variance extracted; VIF =  variance inflation factor.

Two widely accepted types of validity must be established: convergent validity and discriminant validity [90]. Based on its description, the average variance extracted [AVE] [89] and composite reliability [CR] [93] were used to evaluate convergent validity. The results in Table 1 show that the values of each construct were higher than the cut-off point of 0.5 for AVE and 0.7 for CR; Hence, the assumption of convergent validity is met.

While for discriminant validity, the Fornell-Larker and heterotrait-monotrait [HMTM] criteria were used [95]. The HMTM ratio of correlations to be significant must be less than one [96]. Table 2 indicates that this criterion is met. Further, we used the square root of the AVE to measure discriminant validity [93]. In this sense, the square root of the AVE for each construct must be greater than the correlations between constructs [89]. Table 3 reports that the assumed discriminant validity is met because the square root of the AVE of each construct [in bold diagonal] is greater than the correlations between other constructs.

**Table 2:** Result of discriminant validity [HTMT2].

Constructs	IQ	PU	PE	SAT	CI	IVD
IQ						
PU	**0.801**					
PE	0.740	**0.752**				
SAT	0.697	0.744	**0.767**			
CI	0.726	0.723	0.721	**0.718**		
IVD	0.550	0.590	0.537	0.509	**0.599**	

IQ =  information quality; PU =  Perceived usefulness; PE =  perceived enjoyment; SAT =  Satisfaction; CI =  continuance intention; IVD =  intention to visit destination. Los números muestran la proporción de HTMT para dos construcciones.

**Table 3:** Result of discriminant validity [Fornell-Larcker].

Constructs	IQ	PU	PE	SAT	CI	IVD
IQ	**0.880**					
PU	0.764^***^	**0.923**				
PE	0.704^***^	0.717^***^	**0.934**			
SAT	0.663^***^	0.705^***^	0.730^***^	**0.867**		
CI	0.699^***^	0.697^***^	0.694^***^	0.684^***^	**0.951**	
IVD	0.519^***^	0.559^***^	0.506^***^	0.480^***^	0.571^***^	**0.884**

IQ =  information quality; PU =  Perceived usefulness; PE =  perceived enjoyment; SAT =  Satisfaction; CI =  continuance intention; IVD =  intention to visit destination visit; *** =  P values for correlations = <0.001.

Finally, before analyzing the structural model, we evaluate the general model. The model’s fit can be assessed by applying the standardized root mean square residual [SRMR], which must be below 0.08 [97]. In our research, the value of SRMR is 0.052, which suggests a good fit for the measurement model.

### 4.2. Examining the structural model

**Fig 1:**
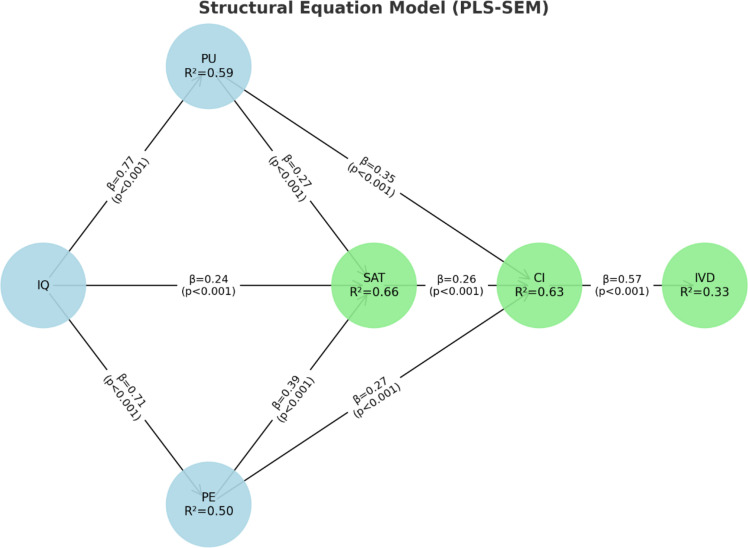
Structural model.

Upon confirming a measurement model that meets the reliability and validity assumptions, the next step was to examine the structural model using the sign, size, and significance of the path coefficients [91] to statistically verify the theoretically established paths and validate the developed hypotheses. Additionally, to indicate the explanatory power of the model, R2. Values were calculated for the five endogenous [dependent] constructs. Table 4 and Fig 1 show the results of the structural model, including the path coefficients and R2.

**Table 4:** Result: Structural model.

Hypotheses	Paths	Path coefficient	T statistics	Confidential internal 95%	Effect size *f*^2^	Remarks
H1	IQ → PU	0.77^***^	14.619	[0.664, 0.869]	0.588	Yes
H2	IQ → SAT	0.24^***^	13.352	[0.125, 0.349]	0.169	Yes
H3	IQ → PE	0.71^***^	4.153	[0.603, 0.811]	0.500	Yes
H4	PU → SAT	0.27^***^	4.812	[0.162, 0.384]	0.199	Yes
H5	PE → SAT	0.39^***^	6.983	[0.280, 0.498]	0.292	Yes
H6	PU → CI	0.35^***^	6.234	[0.240, 0.460]	0.253	Yes
H7	SAT → CI	0.26^***^	4.618	[0.151, 0.374]	0.185	Yes
H8	PE → CI	0.27^***^	6.234	[0.155, 0.378]	0.190	Yes
H9	CI → IVD	0.57^***^	10.572	[0.466, 0.678]	0.327	Yes

***p <0.001.

The model fit indicators obtained from WarpPLS 8.0 demonstrate a robust model with strong predictive validity and reliability. The Average Path Coefficient (APC) is 0.309 (P <  0.001), and the Average R-squared (ARS) is 0.468 (P <  0.001), with an Average Adjusted R-squared (AARS) of 0.465 (P <  0.001). The Average Block VIF (AVIF) is 2.084, and the Average Full Collinearity VIF (AFVIF) is 2.343, both well within acceptable limits (<= 5, ideally <= 3.3). The Tenenhaus GoF (GoF) is 0.611, indicating a large effect size. Additionally, the Simpson’s Paradox Ratio (SPR) is 0.857, the R-squared Contribution Ratio (RSCR) is 0.994, the Statistical Suppression Ratio (SSR) is 1.000, and the Nonlinear Bivariate Causality Direction Ratio (NLBCDR) is 1.000, all meeting or exceeding their respective acceptable thresholds. These indicators collectively affirm the robustness and reliability of the structural model.

Concerning the influences of the different constructs in chatbot technology, first of all, the information quality influences the perceived usefulness of tourists [β=0.77; p˂0.001], user satisfaction [β=0.24; p ˂0.001] and perceived enjoyment [β=0.71; p˂0.001]. These results are statistically significant and favor H1, H2, and H3. Therefore, the first three hypotheses are corroborated. Secondly, the effect of perceived usefulness [β=0.27; p˂0.001] and perceived enjoyment on satisfaction [β=0.39; p˂0.001] has a positive impact; therefore, H4 and H6 are accepted. Likewise, the effect of PU [β=0.35; p˂0.001] and PE [β=0.27; p˂0.001] on the intention to continue using the chatbot tool are positive and significant, which corroborates H5 and H7. Also, we demonstrate that user satisfaction is an antecedent in the intention to continue using a chatbot [β=0.26; p˂0.001], corroborating H8. Finally, the intention to continue is an antecedent that will influence the tourist to visit a destination [β=0.57; p˂0.001]; therefore, H9 is supported (see Table 4).

## 5. Discussion

Based on TAM, ECM, and ISSM theories, this research has provided interesting results, for the first time in the trip planning phase, on the complex mechanisms of user satisfaction, the intention to continue using the chatbot, and the decision to visit a destination after the experience of using it.

The results obtained in this study have confirmed all the proposed hypotheses. Each of the theorized relationships between the constructs, such as the influence of information quality on user satisfaction, perceived usefulness, and enjoyment, as well as the relationship between these factors and the intention to continue using the chatbot, has been empirically validated. Furthermore, it has been corroborated that the intention to continue using the chatbot significantly predicts the intention to visit the destination, aligning with the underlying theories proposed in the conceptual model.

Firstly, the informativeness of AI-powered chatbot conversational agents positively affects user satisfaction, enjoyment, and perceived usefulness. This coincides with previous studies, which have shown that the provision of sufficient, accurate, truthful, and updated information generates user satisfaction [56] and that IQ is an essential construct that creates satisfaction in the use of chatbots [67]. The study’s results also demonstrate that the chatbot’s quality information improves the perceived sense of enjoyment and usefulness of the tool, thus affecting the perception of the tourist, who will consider the chatbot easier and more fun and will increase their satisfaction in the trip planning.

Secondly, the results have shown that perceived enjoyment and usefulness are psychological and emotional factors influencing the user’s decision to continue using chatbots. This is in line with previous studies, which had shown that the perceived usefulness of a technological tool generates satisfaction and the intention to persist and use, but they were not studies focused on chatbots [58,59,61].

Thirdly, the study shows that a rewarding, enjoyable, and fun experience in human-chatbot interaction increases the likelihood that users will continue using chatbots and use them again. This corroborates previous studies, which showed that perceived enjoyment predicts the intention to use technological agents and chatbots [65–68], although these studies did not focus on trip planning. Thus, tourists consider it a valuable tool when it can help them complete their tasks and objectives in organizing trips. This belief makes tourists more likely to trust it and use it to plan their trips.

Finally, the results indicate that the intention to continue using an AI-powered tourism chatbot strongly predicts tourists’ intention to visit a destination. This finding suggests that the more frequently the chatbot is used when searching for information about a destination, the greater the likelihood it will decide to visit it. These results are in line with previous studies, which demonstrated that the intention to continue using digital platforms [75] or technological tools [76,77] increase decisions to visit destinations. However, the contribution of this study is to have demonstrated it in the use of chatbots and travel planning.

### 5.1. Theoretical implications

This study’s theoretical and methodological contribution is to demonstrate the suitability of certain theoretical models [TAM, ECM, and ISSM] for research on chatbots of tourist destinations. Previous studies have applied TAM, ECM, and ISSM models to the study of various tourism technologies [35,40,41,47,48], but our study has shown that these models are very useful to analyze destination chatbots in the context of travel planning, to examine the crucial factors that predict a satisfactory experience, continued behavior in the use of technology and the tourist’s subsequent intention to visit.

Firstly, our research confirms that the informativeness of chatbots, characterized by the accuracy and relevance of the information they provide, is a key driver of user satisfaction, enjoyment, and perceived usefulness, in line with previous studies [65,72,73]. This aligns with the Technology Acceptance Model [TAM], which posits that the quality of information directly influences users’ perceptions of technology. Our results highlighted the importance of information quality [IQ] as a critical factor in creating positive user experiences with chatbots. The improved perception of usefulness and enjoyment due to high-quality information supports the theoretical constructs of TAM and highlights the role of informativeness in enhancing user engagement.

Secondly, the study reveals that perceived enjoyment and usefulness are significant psychological factors affecting users’ intention to continue using chatbots. This finding reinforces the theoretical framework of the Expectancy Confirmation Model (ECM), which suggests that perceived usefulness and enjoyment contribute to user satisfaction and continued use. Although prior research has focused on general technological tools, our study explicitly demonstrates how these constructs apply to chatbots, thereby expanding the theoretical application of ECM in travel planning.

Thirdly, our findings show that an engaging and enjoyable interaction with chatbots increases users’ likelihood of continued use, as previous studies have shown [74]. This outcome aligns with the Information Systems Success Model (ISSM), which emphasizes the importance of user satisfaction in predicting future use. The study extends the ISSM framework by demonstrating that perceived enjoyment significantly predicts continued use, even in specific contexts like travel planning. This underscores the value of creating positive user experiences to encourage sustained engagement with chatbots.

The other significant theoretical contribution is covering a gap in studies on the subject. Since the emergence of ChatGPT and the development of AI, technologies have increasingly been used in travel planning. But even though studies on chatbots have increased [17,18,98], there is practically no research on chatbots in travel planning and their influence on decisions to visit a tourist destination. This study contributes to filling this gap.

### 5.2. Managerial implications

The study analyzes how potential tourists perceive the conversational style of a chatbot in terms of quality of information, perceived usefulness, enjoyment of the tool, and their subsequent satisfaction and intention to continue using it. By exploring a diversity of cognitive and affective constructs, scholars and designers of these technologies can understand how tourists experience psychological changes and decide to select and visit a destination based on their experience interacting with the chatbot.

Thus, destinations and chatbot designers can improve the AI capabilities of this tool in three ways: First, chatbots must take care of and improve their contingency capacity so that the conversation is compelling, dynamic, and interesting, provides them with fair and quality information they need at every moment of trip planning. Secondly, the chatbot must be perceived as easy to use and very useful so that potential tourists continue to use it during planning.

Furthermore, improving the chatbot’s conversational quality to make interactions enjoyable and engaging can significantly impact user retention. A chatbot that combines effective information delivery with a pleasant user experience is more likely to foster trust and satisfaction among tourists. This approach can lead to increased usage and positive word-of-mouth, ultimately influencing travel decisions.

The strong relationship between the intention to continue using chatbots and the decision to visit a destination highlights the strategic importance of chatbots in the tourism industry. By leveraging chatbots as tools to facilitate travel planning, destinations can enhance their attractiveness and encourage visits. The ability of chatbots to provide personalized and engaging interactions can be a key differentiator in the competitive travel market.

Finally, the friendly, warm, empathetic, and emotional tone in conversation styles, which generate fun conversations, make chatbots emulate humans and establish emotional connections with users, facilitating communication. Consequently, taking advantage of the digital footprint of user interactions is crucial to offering personalized services based on extensive data analysis of conversations.

## 6. Conclusion

In conclusion, the results have shown the influence of information quality on user satisfaction, perceived usefulness and enjoyment, as well as the relationship between these factors and the intention to continue using the chatbot. Furthermore, it has been confirmed that the intention to continue using the chatbot significantly predicts the intention to visit the destination.

The study’s theoretical and methodological contribution has been to demonstrate the suitability of the theoretical models used [TAM, ECM, and ISSM] for the analysis on destination chatbots and to cover a gap in studies on the subject. The results also have shown that perceived enjoyment and usefulness are factors that influence user’s decisions to continue using chatbots; and an engaging and enjoyable interaction with chatbots increases users’ continuance intention.

Therefore, the study will help scholars and designers of these technologies to understand how tourists experience the interaction with chatbots and decide to select and visit a destination based on it.

Conversational agents are a disruptive technology that is changing how people communicate and interact with tourists. AI-powered conversational agents like chatGPT Bard are being used in travel planning. Destinations and tourism service providers are adapting these technologies to improve the tourist experience before the trip. Destinations are even beginning to develop human-chatbot tour guides powered by AI, which can imitate human guides’ behavior, allowing a visual experience for the tourist. Therefore, the future of tourism communication will be highly influenced by voice and data conversational agents needing continuous optimization to improve communication. However, what is essential for this to happen has a lot to do with enhancing the information provided to generate user satisfaction and their intention to continue using these technologies.

### 6.1. Limitations

One of the main limitations of this study is the homogeneity of the sample, which is predominantly composed of young university students under 25 years old, many of whom had prior experience using chatbots. This may limit the generalization of the findings to broader populations, particularly to older individuals or those who have not previously interacted with chatbots. Future studies should aim to include a more diverse range of participants, both in terms of age and experience, to assess whether the results hold across different demographic groups. It could also be applied to users with varying sociocultural characteristics, including those resistant to innovation or with technological asymmetries, in order to compare tourist behavior based on cultural background.

Another limitation relates to the functionality of the chatbot used in this study. While it offers several hotel options and integrates links to online travel agencies like TripAdvisor, it does not create personalized travel packages nor can it make reservations for events or tourist attractions. This limited functionality may have impacted user satisfaction and the intention to continue using the chatbot. More research is needed to evaluate the impact of more advanced chatbot features, such as personalized recommendations, on tourist behavior and satisfaction.

## Appendix 1

### Indicators for constructs

**Table:** 

Construct	Indicators	Sources
**Information quality [IQ]**	IQ1: Provided sufficient information	[85]
	IQ2: I get the information I need on time	
	IQ3: Provided information in a useful format	
	IQ4: Provided accurate information	
	IQ5: Provided precise information	
	IQ6: Provided up-to-date information	
	IQ7: Provided reliable information	
**Perceived usefulness [PU]**	PU1: The chatbot helped plan my trip to Quito	[69]
	PU2: The chatbot application makes the city tour useful	
	PU3: The chatbot application is an effective way to get a Quito city tour	
	PU4: I use the chatbot to get better access to information on the Quito city	
	PU5: Overall, I find using the chatbot application useful	
**Perceived enjoyment [PE]**	PE1: I feel that using this chatbot is fun.	[67]
	PE2: I feel that using this chatbot is exciting.	
	PE3: I feel that using this chatbot is enjoyable.	
	PE4: I feel that using this chatbot is interesting.	
**Satisfaction [SAT]**	[after chatting with the chatbot]	[86]
	SAT1: I am satisfied with the chatbot experience	[63]
	SAT2: I am pleased with the chatbot.	
	SAT3: The chatbot exceeded my expectations	
	SAT4: The chatbot is close to my ideal technology	
	SAT5: Overall, I am satisfied with the chatbot application	
**Continuance intention [CI]**	CI1: I will try to use this chatbot in the planning of my trip to Quito	[67]
	CI2: I will frequently use the chatbot “Quito guide” in the future	
	CI3: I intend to continue using the chatbot over other technological tools	
	CI4: In my case, the use of this chatbot service may increase.	
	CI5: I strongly recommend that others use the chatbot “Quito guide.”	
**Intention visit destination [IVD]**	IVD1: I intend to visit Quito city frequently	[87]
	IVD2: I will visit Quito city	
	IVD3: I want to recommend Quito City to others	
	IVD4: I will likely visit Quito in the future	
	IVD5: I plan to visit Quito in the future	
